# Supplementary material for: InSe as a case between 3D and 2D layered crystals for excitons
Source: Nat Commun. 2019 Aug 2;10:3479. doi: 10.1038/s41467-019-11487-0 (PMC6677765; doi:10.1038/s41467-019-11487-0)
Supplement: Supplementary file 1 — Supplementary Information [file 41467_2019_11487_MOESM1_ESM.pdf]

InSe as a case between 3D and 2D layered crystals for excitons

T.V. Shubina, et al.

SUPPLEMENTARY INFORMATION

SUPPLEMENTARY FIGURES

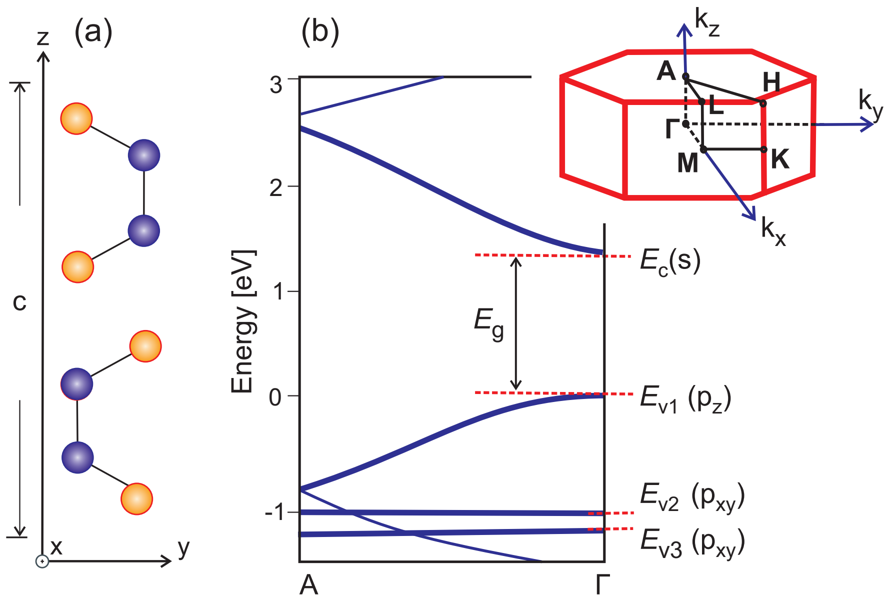

SUPPLEMENTARY FIGURE 1: (a) Stacking of β-polytype of InSe, where the blue spheres mark In and yellow ones - Se; z-axis is directed normal to the tetralayers, along the c lattice parameter. (b) Schematic of the InSe band structure near the optical gap (without spin-orbit interaction). The inset shows the first Brillouin zone of β-InSe.

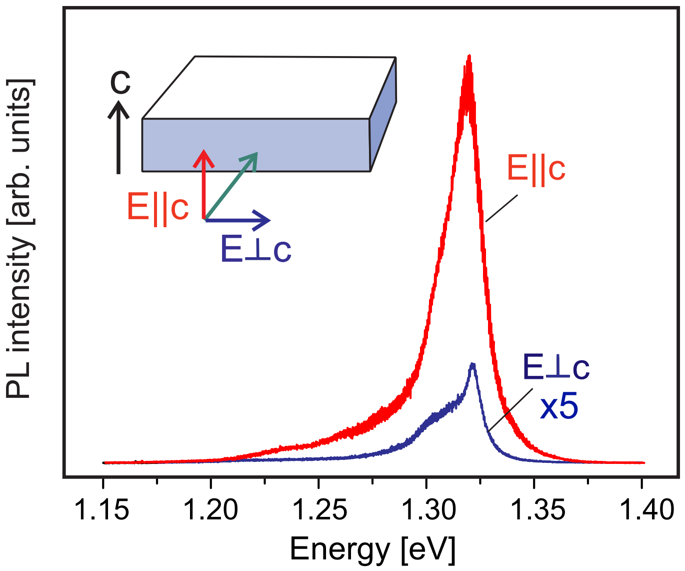

SUPPLEMENTARY FIGURE 2: Polarized excitonic emission measured from the cleaved edge of an InSe crystal at 80 K in two polarizations $E \parallel c$ and $E \perp c$, as shown in the inset. The energy of the PL peak is consistent with the 80 K data shown in Fig. 3 in the main text.

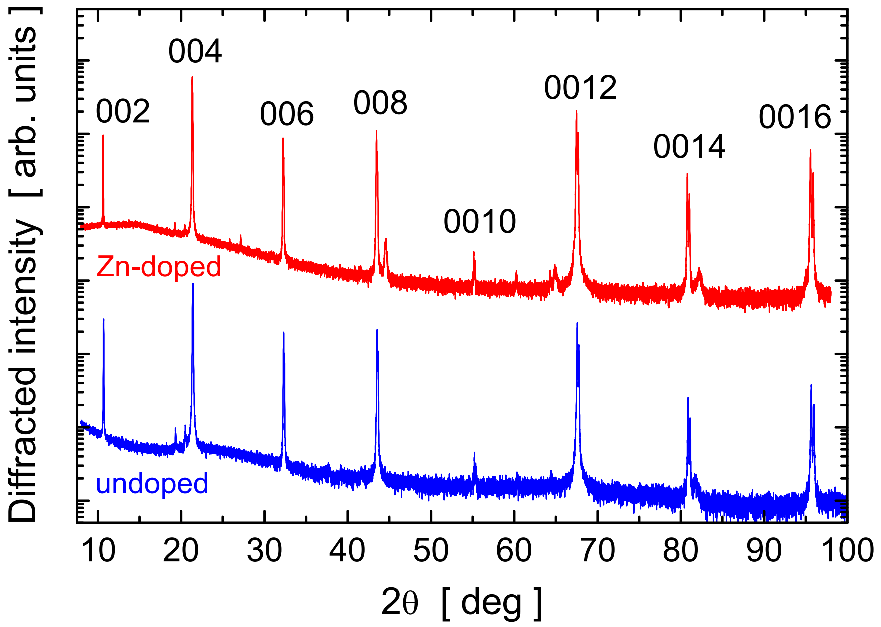

SUPPLEMENTARY FIGURE 3: $\theta/2\theta$ XRD spectra of the undoped and Zn-doped bulk InSe samples.

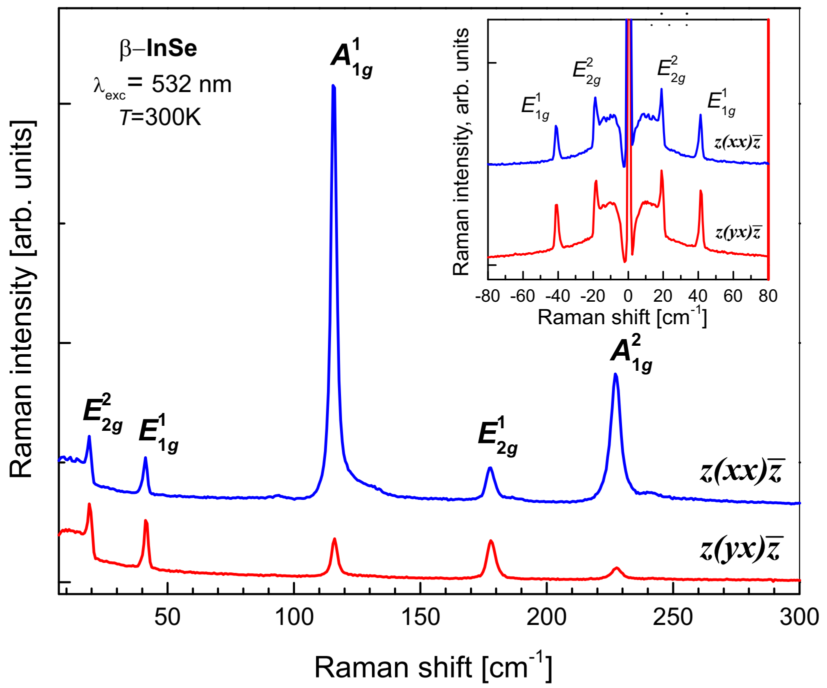

SUPPLEMENTARY FIGURE 4: Polarized Raman spectra of the undoped bulk β-InSe. The z direction in the scattering configurations is parallel to the optical c axis. The inset shows Stokes and anti-Stokes peaks of rigid-layer modes in the low-frequency region of the spectra.

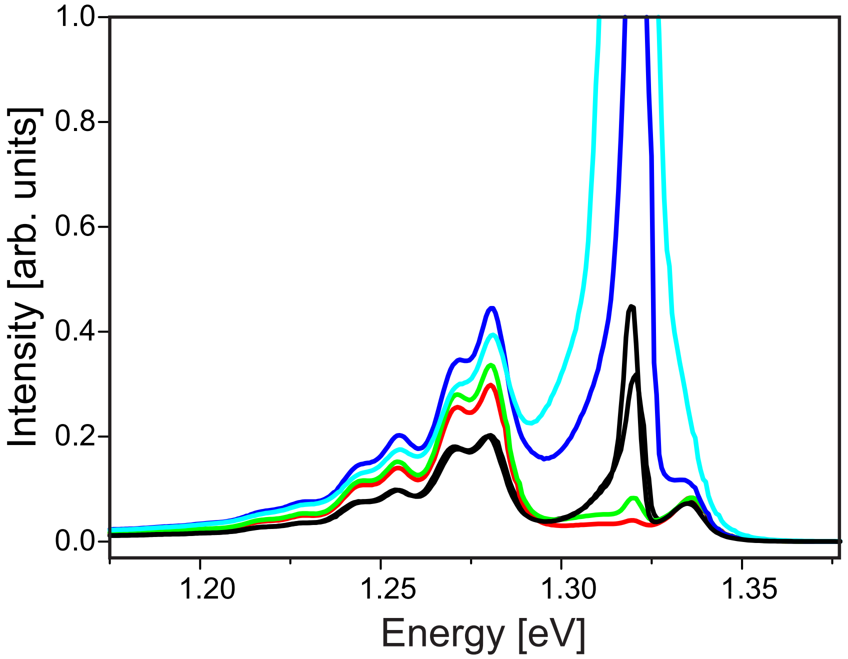

SUPPLEMENTARY FIGURE 5: Spatial dependence of the PL spectra at $T = 10$ K and ~ 0.3 $MWcm^{-2}$ laser power for the undoped InSe sample. The distance between two adjacent spots is ~ 1 mm.

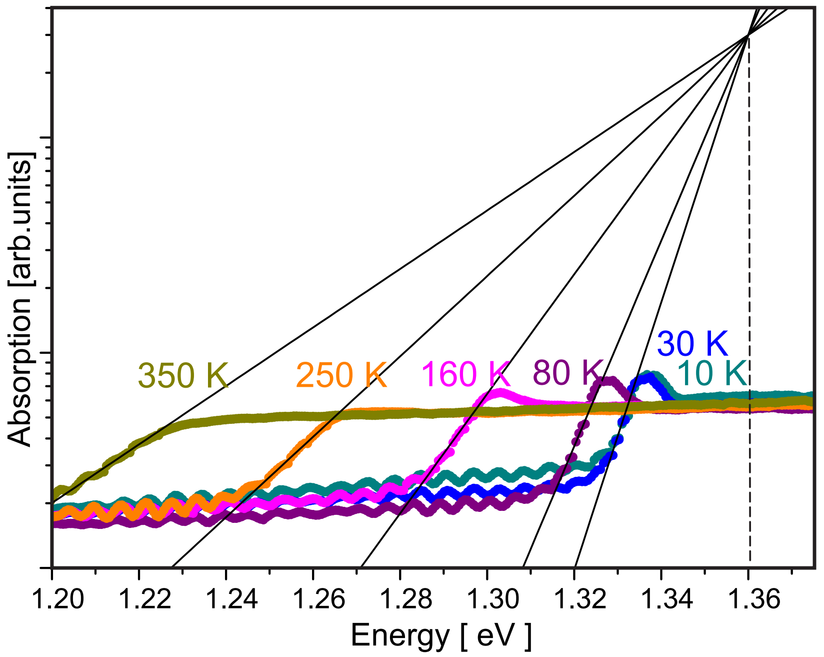

SUPPLEMENTARY FIGURE 6: Absorption spectra (log scale) measured in the undoped InSe at different temperatures shown together with the fittings of the Urbach tails.

SUPPLEMENTARY TABLES

SUPPLEMENTARY TABLE 1: Parametrization of the excitons in InSe

Title	$m_{xx,e}/m_0$	$m_{zz,e}/m_0$	$m_{xx,h}/m_0$	$m_{zz,h}/m_0$	μ_{xx}/m_0	μ_{zz}/m_0	χ	Ref.
I	0.18	0.1	5	0.11	0.173	0.055	3.93	[1]
II	0.12	0.03	3.1	0.03	0.115	0.0155	9.27	[2]
III	0.14	0.08	0.73	0.17	0.118	0.055	2.68	[3,4]

SUPPLEMENTARY TABLE 2: Binding energies E_{b} of ground $1S$ and excited $2S$,$3s$ and $2P$ exciton states

Title	E_{b}^{1s}, meV	E_{b}^{2s}, meV	E_{b}^{3s}, meV	E_{b}^{2p}, meV
I	27.5	7.2	3.3	3.3
II	12.2	3.3	1	1.5
III	21.7	5.6	2.6	3.2

SUPPLEMENTARY TABLE 3: Low-temperature energies of exciton series and exciton binding energy of InSe and GaSe. The exciton binding energies R_{X} is calculated using the hydrogen-like model of isotropic crystal for the $\Delta_{2,1} = E_{n=2} - E_{n=1}$ and $\Delta_{3,2} = E_{n=3} - E_{n=2}$. The temperature of measurements is indicated to account for the possible temperature shift.

Material	$E_{n=1}$, meV	$E_{n=2}$, meV	$E_{n=3}$, meV	$R_{\mathrm{X}}(\Delta_{2,1})$, meV	$R_{\mathrm{X}}(\Delta_{3,2})$, meV	T, Refs.
InSe	1338.3	1349.2	1352	14.5	20.2	1.6 K[5]
InSe	1338.5	1350	1352.9	15.3	20.9	4.2 K[6]
InSe	1320	1347	-	37	-	80 K,[7]
GaSe	2111.6	2126.6	2129.3	20	19.4	4.2 K[6]
GaSe	2110	2124.6	2127.4	19.5	20.2	10 K[8]

SUPPLEMENTARY NOTES

SUPPLEMENTARY NOTE 1 : Band structure and polytypes

The band structure of bulk InSe was computed numerically many times using different theoretical methods[2,9–15]. It was also investigated experimentally using photoluminescence (PL) spectroscopy under high pressure, as well as angle resolved photoemission spectroscopy (ARPES)[12,16,17]. InSe has three principal polytypes: the rhombohedral γ-polytype (C_{3v}^5 space group), hexagonal β-polytype (D_{6h}^4), and hexagonal ϵ-polytype (D_{3h}^1)[18]. It is well established that γ- and β-polytypes have direct band structure, which is rather important for optoelectronic applications. They have nearly identical values of bandgaps at ambient conditions: 1.2829 and 1.2931 eV[16,19]. On the contrary, the ϵ-polytype is assumed to have indirect band structure with a higher bandgap value around 1.4 eV[20]. The excitonic PL at ~1.3 eV reported in this work excludes the studied samples to be the ϵ polytype. The structural characterization described in Supplementary Methods 1 and 2 has demonstrated that they most likely belong to the β polytype, whose band structure has been analyzed in several papers[2,21]. We present in Supplementary Figure 1 the tetralayer arrangement and the schematic of the electronic band structure for the β-InSe in a vicinity of optical gap, where the characteristic type of states (s, p_z, p_{xy}) is shown for each band.

SUPPLEMENTARY NOTE 2 : Selection rules of optical transitions

Following Ref.[22], we can construct the direct dipole transitions in the β-InSe of the D_{6h}^4 symmetry from the Bloch orbits Γ_1^+ and Γ_2^- taking into account spin by going into the double group representation

$$\overline{D}_{6h}^4 = D_{6h}^4 \times D^{(1/2)}, \tag{1}$$

where $D^{(1/2)}$ is the two-dimensional representation of the Pauli spinors. Consistently, from Koster's tables[23]: $\Gamma_1^+ \times \Gamma_7^\pm = \Gamma_7^\pm$ and $\Gamma_2^- \times \Gamma_7^\pm = \Gamma_7^\mp$. The product $\Gamma_7^\pm \times \Gamma_7^\mp = \Gamma_1^- + \Gamma_2^- + \Gamma_5^-$ contains both $E \parallel c$ (Γ_1^-) and $E \perp c$ (Γ_5^-) optically allowed representations. The Γ_2 states are dipole-forbidden. The optical transition Γ_5^- comprising $x \pm iy$ components occurs possible only when both spin and orbit are coupled. This coupling gives rise to a mixing of the highest valence band with the lower-lying bands $E_{v2,v3}$ (see Supplementary Figure 1). Interband mixing between conduction bands can be neglected because their separation is much higher. Thus the transitions involving states of s-symmetry are only partly allowed for $E \perp c$, while they are fully allowed for light polarization $E \parallel c$ axis.

The excitonic states near the optical gap in Γ point are mediated by these selection rules. In the weak spin-orbit coupling regime, like in our case when the $E_{v2,v3}$ bands are distant, the singlet component of excitons originating from Γ_1 is strong in $E \parallel c$, while the triplet component Γ_5 interacts weakly with light when $E \perp c$. This situation in relevance to the optical absorption is illustrated by Fig. 5a in the main text. Thus, the exciton transitions at $E \perp c$ in InSe cannot be considered neither allowed (bright) nor forbidden (dark) in the usual sense of these terms. We have to claim them as partly allowed (gray).

Direct confirmation of the impact of the selection rules on optical properties has been obtained by measuring the polarized PL from the cleaved edge of InSe crystal in two polarizations: $E \parallel c$ and $E \perp c$. It turns out to be that their intensities differ more than by the order of magnitude (see Supplementary Figure 2). The possibility to observe such symmetry-related phenomenon also indicates the high quality of the studied samples.

SUPPLEMENTARY NOTE 3 : Anisotropic model for excitons in InSe

We treat an exciton in InSe in the framework of the effective mass approach with anisotropic mass and anisotropic dielectric function[24,25]. The respective Hamiltonian reads as

$$\widehat{H}_{ex} = -\left[\frac{\partial^2}{\partial x^2} + \frac{\partial^2}{\partial y^2} + \frac{\mu_{xx}}{\mu_{zz}}\frac{\partial^2}{\partial z^2}\right] - \frac{2}{\sqrt{x^2 + y^2 + \kappa_{xx}/\kappa_{zz} z^2}}. \tag{2}$$

The effective Rydberg

$$Ry = \frac{\mu_{xx} e^4}{2\hbar^2 \kappa_{xx}\kappa_{zz}}$$

and Bohr radius

$$a_{\rm B} = \frac{\sqrt{\kappa_{xx}\kappa_{zz}}\hbar^2}{\mu_{xx} e^2}$$

were used as energy and length units. Here x, y and z are Cartesian coordinates chosen along the crystal axes. The z-axis is directed along c-axis, μ_{ii} and κ_{ii} are the components of the reduced exciton mass and dielectric constant tensors, $\mu_{ii}^{-1} = m_{ii,e}^{-1} + m_{ii,h}^{-1}$, where $m_{ii,e}$ and $m_{ii,h}$ are the components of electron and hole effective masses tensors, respectively. The relations $\mu_{xx} = \mu_{yy} \neq \mu_{zz}$ and $\kappa_{xx} = \kappa_{yy} \neq \kappa_{zz}$ are assumed.

In InSe, the charge carriers effective masses as well as the exciton reduced mass are smaller along the z-axis than in the perpendicular plane so the exciton could has somewhat 1D-like character. However, the parameter which actually control the exciton wave function is $\chi = \kappa_\perp\mu_\perp/\kappa_\parallel\mu_\parallel$. In our calculations, we have used three parametrizations for effective masses as in Ref.[18] shown in Supplementary Table 1, which are two strongly different theoretical sets (I and II) in order to estimate the influence of parameters dispersion and one more (III) based on experimental data obtained by alternative methods, such as the study of acceptor-bound exciton states under pressure[4] and cyclotron-resonance studies of electron masses[3]. The latter data are close to those obtained by magnetoabsorption spectroscopy[26]. For all cases, the relative dielectric constants $\kappa_{xx} = 8$, $\kappa_{zz} = 6.4$ as in the Ref.[16] have been taken.

The binding energy $E_{\rm b}$ of ground 1s exciton state (marked as $R_{\rm X}$ in the main text) has been calculated by both variational approach and numerical calculations using the same Hamiltonian to check their consistency. Because we have obtained very good agreement between the obtained results, the developed numerical method was used to calculate the binding energies of excited 2s and 3s exciton states. In addition, we calculate the binding energy of the 2p state. It cannot be observed in the single-photon absorption; two-photons spectroscopy is needed for that. However, being situated closely to 3s, the 2p state can be mixed with it that effects on the possibility to resolve the 3s state. The results for magnetic quantum number $m = 0$ are shown in Supplementary Table 2.

We notice the good agreement between $E_{\rm b}^{1S} = 21.7$ meV, calculated using the experimental data obtained by the alternative methods (see Supplementary Table 2), and our result $R_{\rm X} \simeq 22$ meV, derived from the experiments on exciton scattering (see main text). The corresponding χ value varies from 2.68 (see Supplementary Table 1) up to 2.86 when the parameters from Ref.[26] was used. The dependence of $E_{\rm b}^{1S}$ on the anisotropy parameter χ, calculated in the validity range of the effective mass approach ($0.1 < \chi < 5$), is presented in Fig. 5b of the main text.

SUPPLEMENTARY NOTE 4 : Analysis of published data on exciton energies

We have analysed the published data on the exciton binding energy $R_{\rm X}$ in InSe and GaSe. The data presented in Supplementary Table 3 show that in GaSe the values obtained from the differences $\Delta_{2,1} = E_{n=2} - E_{n=1}$ and $\Delta_{3,2} = E_{n=3} - E_{n=2}$ agree with each other. On the contrary, in InSe such an agreement is absent. The $R_{\rm X}$ derived from the $\Delta_{3,2}$ value is around 21 meV that is markedly higher than the $\Delta_{2,1}$ one equal to $14 - 15$ meV. We should note that this inconsistency does not allow one to declare the 3D character of excitons in InSe.

The outstandingly high $R_{\rm X}$ magnitude obtained by Andriyashik et al.[7] may be related to the attribution of the Sommerfeld peak as the first excited state. That is why their bandgap value reasonably correlates with our value (1.36 eV), taking into account the temperature-induced shift (they performed the measurements at 80 K). This analysis is very important in the context of this paper, because the seeming applicability of the 3D model used was a crucial argument in favor of the 3D character of excitons in InSe.

SUPPLEMENTARY METHODS

SUPPLEMENTARY METHOD 1 : X-ray diffraction

Supplementary Figure 3 shows the $\theta/2\theta$ scans of the undoped and Zn-doped InSe bulk samples. The periodic peaks indicate a perfect stacking of the layers along the z-axis for each crystal. The c lattice parameter is obtained from the linear dependence of the interplanar distance d vs inverse integer index (not shown). The values are $c = 16.542$ $\AA$ and 16.650 $\AA$ for the undoped and p-type crystals, respectively. This could correspond the β-InSe[14] because the rhombohedral InSe has the lattice parameter about $c = 24.95$ $\AA$ [2,27]. In order to completely define the polytype the crystal has been oriented on purpose for the detection of an asymmetric line. The (107) plane specific to the hexagonal structure has indeed been observed for the expected crystal orientation (not shown here). This signature rules out other stacking orders.

SUPPLEMENTARY METHOD 2 : Raman studies

The Raman measurements were performed at room temperature using a T64000 (Horiba Jobin-Yvon) spectrometer equipped with a confocal microscope. The line at 532 nm (2.33 eV) of Nd:YAG laser (Torus, Laser Quantum, Inc.) was used as the excitation source. The laser power on the sample was as low as 25 μW in a spot size of $\sim$1 μm. Supplementary Figure 4 shows the typical polarized Raman spectra of InSe obtained in two backscattering geometries: $z(xx)\bar{z}$ and $z(xy)\bar{z}$. Here, the z direction is parallel to the c axis, and x and y are mutually orthogonal and oriented in an arbitrary manner in the layer plane. Five narrow Raman lines have been observed at 19.0, 41.3, 115.4, 177.2, and 227.1 cm^{-1}. The most important finding is the observation of the low-frequency interlayer mode (vibration of tetralayer as a whole) at 19.0 cm^{-1}, very close to the laser excitation line. This is the main signature of the β- or ϵ-polytype of InSe. The primitive unit cell of γ-polytype contains only one InSe layer[18]. Therefore all optical modes are intralayer ones and the interlayer vibrations should not be observed in the first-order Raman spectra of γ-InSe. In our case, ϵ-InSe polytype should be also excluded because of the registered PL energy which is characteristic for the β-InSe. Small FWHM of Raman lines and a very good agreement with the Raman selection rules[18,28] clearly demonstrate that the studied samples can be classified as the high-quality β-InSe. This conclusion is fully consistent with the X-ray data and the observation of polarized PL satisfying the exciton selection rules.

SUPPLEMENTARY METHOD 3 : Optical measurements

The nonlinear optical processes can be studied in two different experimental geometries: i) incident excitation to the sample surface and detection of the emission from the surface (”backscattering geometry”)[29,30]; ii) excitation to the surface but detection from a cleaved facet[31]. On one hand, the waveguiding effect (and symmetry-enhanced PL intensity), characteristic for the later geometry, can promote the stimulated emission. On the other hand, it leads to an additional red-shift and a broadening of the emission lines. We used the first geometry in our experiments.

The low-intensity photoluminescence (PL) was measured using a continuous 15 mW red laser (650 nm). High pump power was obtained with a Q-switched green laser (532 nm) with a pulse duration of 15 ns and a repetition rate of 1 kHz, which corresponds to a maximal power of 100 W per laser peak. The pump intensity was varied using the combinations of neutral filters. The PL was detected using a Hamamatsu InGaAs photomultiplier cooled at 77 K. The integration of pulsed response over time was performed by a lock-in amplifier.

A reasonable estimate of the laser spot diameter of $\sim$200 μm leads to a maximal power density of $\sim$ 0.3 MWcm^{-2}. Note that such a density is significantly lower than the 6 MWcm^{-2} used in the previous experiments on stimulated emission in InSe[32].

The intensity of emission peaks measured at $T = 10$ K and for maximal laser power is strongly dependent on the position of the laser spot on the surface of the InSe crystal (see Supplementary Figure 5). The strong dependence of the peak intensities on the laser spot location results either from the softness of the InSe crystal, which can be

easily damaged during cleavage, or from interlayer defects[33]. Both sources lead to spatially inhomogeneous intensity of optical response[34]. At the same time, the energy positions of the peaks are constant within a ± 0.5 meV limit.

The transmission measurements presented here were performed on a thinner (~ 40 μm) InSe sample with back illumination with a halogen lamp through a 1 mm diameter pinhole. All samples were mounted on the cold finger of a closed cycle helium cryostat with the possibility of temperature variation in the $10 - 350$ K range.

SUPPLEMENTARY METHOD 4 : Defect-related emission

The defect related lines below the P-band exhibit intensity saturating with the power increase. Besides, they quench fast with the temperature rise due to the carrier delocalization from bounding centers. First line marked by (*) was reported in many previous studies as a donor-related transition[34,35]. It was proposed that this transition could be related to an indirect exciton[36]. However the currently accepted band structure of InSe (see Supplementary Figure 1) rules out this suggestion definitely. In our studies, this assignment is supported by its arising in the naturally n-doped InSe and quenching in the p-doped structures. This line is not resolved in spectra in Supplementary Figure 5 measured with high excitation power due to its saturation.

Excitons bound to very shallow neutral donors have been reported in InSe around $1.321 - 1.334$ eV but are not detected in our samples[37–39]. The peaks detected at 971.5 nm, 991 nm, and 1013 nm have been assigned previously to donor-acceptor transition[34], to donor-to-band transition in lithium intercalated InSe[38] or excitons bound to donor levels such as V_{Se}[40]. Ferrer-Roca *et al.* interpreted these peaks as a band-to-acceptor recombination and LO-phonon replicas[4]. According to this analysis and assuming the 971.5-peak is the zero-phonon recombination we evaluate the acceptor binding energy to 76 meV. The energy separation between adjacent peaks is almost constant and is of the order of $25 - 27$ meV. This value is in agreement with the 201 and 227 cm^{-1} optical phonons (25 and 28 meV respectively)[20,41,42]. Finally, a broad peak occurs at larger wavelength centered at 1084 nm (1.144 eV) (not shown in the presented PL plots) was previously observed in p-type InSe and attributed to the Zn acceptor level[43,44]. Indeed this PL peak is even more pronounced in the PL spectrum of the Zn-doped InSe sample.

SUPPLEMENTARY METHOD 5 : Temperature dependent photoluminescence and absorption

The temperature dependence of the free exciton energy shown in the inset of Fig. 3 in the main text can be modeled by $E(T) = A - B\coth(\hbar\omega_{\mathrm{ph}}/2k_{\mathrm{B}}T)$. The fit of our data leads to a phonon energy of 13.7 meV, which is in agreement with the 14 meV value reported in Ref.[5] and the 13 meV estimate extracted from our transmission data. At high temperature the FX peak shifts linearly with temperature at a rate of $-2k_{\mathrm{B}}B/\hbar\omega_{\mathrm{ph}} \approx -0.41$ meV/K.

The absorption spectra exhibit the pronounced Urbach tails (see Supplementary Figure 6). Their temperature dependence is given by

$$\alpha(\hbar\omega, T) = \alpha_0 \exp\left[\frac{(\hbar\omega - E_0)}{E_{\mathrm{U}}(T)}\right] \tag{3}$$

where $E_{\mathrm{U}}(T) = k_{\mathrm{B}}T/\sigma$ is the Urbach energy equal to the energy width of the absorption edge. E_0 and α_0 are the coordinates of the convergence point of the Urbach "bundle", which is the crossing point of the fitting lines. It occurs close to 1.36 eV in our samples. The steepness parameter σ depends on the temperature and the energy of phonons involved in the absorption process:

$$\sigma = \sigma_0 \frac{2k_{\mathrm{B}}T}{\hbar\omega_{\mathrm{ph}}} \tanh\frac{\hbar\omega_{\mathrm{ph}}}{2k_{\mathrm{B}}T}, \tag{4}$$

where $\hbar\omega_{\mathrm{ph}}$ is the energy of the phonon involved in the broadening, likely an LO phonon.

We refrain from defining the exact value of E_{g} based on the absorption data, because the 1s absorption peak is smoothed and we do not observe the features of excited levels.

SUPPLEMENTARY REFERENCES

[1] Bourdon, A., Chevy, A. & Besson, L. Band Structure of Indium Selenide. *Proceedings of the 14th International Conference on the Physics of Semiconductors, Edinburgh, Scotland, 4–8 September 1978; Ed. H. Wilson; Institute of Physics and Physical Society: London, UK* **10**, 1371–1374 (1979).

[2] Gomes da Costa, P., Dandrea, R. G., Wallis, R. F. & Balkanski, M. First-principles study of the electronic structure of γ-InSe and β-InSe. *Phys. Rev. B* **48**, 14135 (1993).

[3] Kress-Rogers, E., Nicholas, R. J., Portal, J. C. & Chevy, A. Cyclotron-resonance studies on bulk and two-dimensional conduction electrons in InSe. *Solid State Commun.* **44**, 379–383 (1982).

[4] Ferrer-Roca, C., Segura, A., Andrés, M. V., Pellicer, J. & Muñoz, V. Investigation of nitrogen-related acceptor centers in indium selenide by means of photoluminescence: Determination of the hole effective mass. *Phys. Rev. B* **55**, 6981–6987 (1997).

[5] Camassel, J., Merle, P., Mathieu, H. & Chevy, A. Excitonic absorption edge of indium selenide. *Phys. Rev. B* **17**, 4718–4725 (1978).

[6] Savchuk, A. I. *et al.* Two-dimensional materials as inorganic analogues of graphene: methods of preparation, optical properties and applications. *Proc. SPIE 9809, 12th International Conference on Correlation Optics* **9809**, 98090X (2015).

[7] Andriyashik, M. V., Sakhnovskii, Y. M., Timofeev, V. B. & Yakimova, A. S. Optical Transitions in the Spectra of the Fundamental Absorption and Reflection of InSe Single Crystals. *Phys. Stat. Sol. (b)* **28**, 277–285 (1968).

[8] Toullec, R. L., Piccioli, N. & Chervin, J. C. Optical properties of the band-edge exciton in GaSe crystals at 10 K. *Phys. Rev. B* **22**, 6162–6170 (1980).

[9] McCanny, J. V. & Murray, R. B. The band structures of gallium and indium selenide. *J. Phys. C: Solid State Phys.* **10**, 1211–1222 (1977).

[10] Depeursinge, Y., Doni, E., Girlanda, R., Baldereschi, A. & Maschke, K. Electronic properties of the layer semiconductor InSe. *Solid State Commun.* **27**, 1449–1453 (1978).

[11] Kuroda, N., Munakata, I. & Nishina, Y. Exciton transitions from spin-orbit split off valence bands in layer compound InSe. *Solid State Commun.* **33**, 687–691 (1980).

[12] Manjón, F. J. *et al.* Band structure of indium selenide investigated by intrinsic photoluminescence under high pressure. *Phys. Rev. B* **70**, 125201 (2004).

[13] Olguín, D., Rubio-Ponce, A. & Cantarero, A. Ab initio electronic band structure study of III-VI layered semiconductors. *Eur. Phys. J. B* **86**, 350 (2013).

[14] Rybkovskiy, D. V., Osadchy, A. V. & Obraztsova, E. D. Transition from parabolic to ring-shaped valence band maximum in few-layer GaS, GaSe, and InSe. *Phys. Rev. B* **90**, 235302 (2014).

[15] Sun, Y. *et al.* InSe: a two-dimensional material with strong interlayer coupling. *Nanoscale* **10**, 7991–7998 (2018).

[16] Politano, A. *et al.* Indium selenide: an insight into electronic band structure and surface excitations. *Sci. Rep.* **7**, 3445 (2017).

[17] Henck, H. *et al.* Evidence of direct electronic band gap in two-dimensional van der Waals indium selenide crystals. *Phys. Rev. Materials* **3**, 034004 (2019).

[18] Segura, A. Layered Indium Selenide under High Pressure: A Review. *Crystals* **8**, 206 (2018).

[19] Zelewski, S. J. & Kudrawiec, R. Photoacoustic and modulated reflectance studies of indirect and direct band gap in van der Waals crystals. *Sci. Rep.* **7**, 15365 (2017).

[20] Lei, S. *et al.* Evolution of the electronic band structure and efficient photo-detection in atomic layers of InSe. *ACS Nano* **8**, 1263–1272 (2014).

[21] Sang, D. K. *et al.* Two dimensional β-InSe with layer-dependent properties: Band alignment, work function and optical properties. *Nanomaterials MDPI* **9**, 82 (2019).

[22] Mooser, E. & Schlüter, M. The Band-Gap Excitons in Gallium Selenide. *Nuovo Cimento* **18**, 164–208 (1973).

[23] Koster, G. F., Dimmock, J. O., Wheeler, R. G. & Statz, H. *Properties of the thirty-two point groups* (M.I.T. Press, Cambridge, Massachusetts, 1963).

[24] Devérin, J. The effective mass approximation for excitons in anisotropic crystals. *Il Nuovo Cimento* **LXIII B**, 1–9 (1969).

[25] Baldereschi, A. & Diaz, M. G. Anisotropy of excitons in semiconductors. *Il Nuovo Cimento* **LXVIII B**, 217–229 (1970).

[26] Millot, M., Broto, J.-M., George, S., González, J. & Segura, A. Electronic structure of indium selenide probed by magnetoabsorption spectroscopy under high pressure. *Phys. Rev. B* **81**, 205211 (2010).

[27] Chevy, A., Kuhn, A. & Martin, M.-S. Large InSe monocrystals grown from a non-stoichiometric melt. *J. Crystal Growth* **38**, 118–122 (1977).

[28] Belenkii, G. L. & Stopachinskii, V. B. Electronic and vibrational spectra of III-VI layered semiconductors. *Phys. Usp.* **26**, 497–517 (1983).
[29] Mercier, A. & Voitchovsky, J. P. Exciton-exciton and exciton-carrier scattering in GaSe. *Phys. Rev. B* **11**, 2243–2250 (1975).
[30] Cingolani, R., Ferrara, M. & Lugarà, M. Direct and indirect electron-hole plasmas in gallium selenide. *Phys. Rev. B* **36**, 9589–9594 (1987).
[31] Cingolani, A., Ferrara, M., Lugarà, M. & Lévy, F. Stimulated photoluminescence in indium selenide. *Phys. Rev. B* **25**, 1174–1178 (1982).
[32] Cingolani, R., Ferrara, M., Lugarà, M. & Lévy, F. Optical gain in gallium selenide and indium selenide. *Physica* **105B**, 40 (1981).
[33] Nicholas, R. J., Kress-Rogers, E., Portal, J. C., Galibert, J. & Chevy, A. Two-dimensional behaviour due to electrons bound at defects in InSe. *Surf. Sci.* **113**, 339–346 (1982).
[34] Abha & Warrier, A. V. R. Photoluminescence studies on the layer semiconductor InSe. *J. Appl. Phys.* **53**, 5169–5171 (1982).
[35] Abay, B., Efeoğlu, H. & Yoğurtçu, Y. Low-temperature photoluminescence of n-InSe layer semiconductor crystals. *Mater. Res. Bull.* **33**, 1401–1410 (1998).
[36] Gnatenko, Y. P. & Zhirko, Y. I. Exciton Absorption and Luminescence of Indium Selenide Crystals. *Phys. Status Solidi (b)* **142**, 595–604 (1987).
[37] Brebner, J., Steiner, T. & Thewalt, M. Time-resolved photoluminescence study of InSe. *Solid State Commun.* **56**, 929–931 (1985).
[38] Balkanski, M., Julien, C. & Jouanne, M. Electron and phonon aspects in a lithium intercalated InSe cathode. *J. Power Sources* **20**, 213–219 (1987).
[39] Godzaev, M. O. & Sernelius, B. E. Electron-hole liquid in layered InSe: Comparison of two- and three-dimensional excitonic states. *Phys. Rev. B* **33**, 8568–8581 (1986).
[40] Ho, C.-H. & Y, J. C. Bending Photoluminescence and Surface Photovoltaic Effect on Multilayer InSe 2D Microplate Crystals. *Adv. Opt. Mater.* **3**, 1750–1758 (2015).
[41] Sánchez-Royo, J. F. *et al.* Electronic structure, optical properties, and lattice dynamics in atomically thin indium selenide flakes. *Nano Res.* **7**, 1556–1568 (2014).
[42] Pozo-Zamudio, O. D. *et al.*
[43] Ikari, T., Shigetomi, S., Koga, Y. & Shigetomi, S. Photoluminescence of Zn Doped InSe Single Crystals. *Phys. Stat. Sol. (b)* **103**, K81–K83 (1981).
[44] Shigetomi, S., Ohkubo, H., Ikari, T. & Nakashima, H. Zn-induced impurity levels in layer semiconductor InSe. *J. Appl. Phys.* **66**, 3647–3650 (1989).
